# WISP1 Is Increased in Intestinal Mucosa and Contributes to Inflammatory Cascades in Inflammatory Bowel Disease

**DOI:** 10.1155/2016/3547096

**Published:** 2016-06-15

**Authors:** Qi Zhang, Cuiping Zhang, Xiaoyu Li, Yanan Yu, Kun Liang, Xinzhi Shan, Kun Zhao, Qinghui Niu, Zibin Tian

**Affiliations:** Department of Gastroenterology, The Affiliated Hospital of Qingdao University, Qingdao 266003, China

## Abstract

Inflammatory bowel disease (IBD) is mainly characterized by intestinal tissue damage, which is caused by excessive autoimmune responses poorly controlled by corresponding regulatory mechanisms. WISP1, which belongs to the CCN protein family, is a secreted matricellular protein regulating several inflammatory pathways, such as Wnt/*β*-catenin pathway, and has been reported in several diseases including cancer. Here we examined the expression, regulatory mechanisms, and functions of WISP1 in IBD. WISP1 mRNA and protein expression was upregulated in colonic biopsies and lamina propria mononuclear cells (LPMC) of IBD patients compared with those of healthy controls. Tumor necrosis factor- (TNF-) *α* induced WISP1 expression in LPMC from healthy controls. Consistently, WISP1 mRNA expression was downregulated in colonic biopsies from IBD patients who had achieved clinical remission with infliximab (IFX). Furthermore, WISP1 expression was also found to be increased in colons from 2,4,6-trinitrobenzenesulfonic acid- (TNBS-) induced mice compared with those from control mice. Further studies confirmed that administration of rWISP1 could aggravate TNBS-induced colitis in vivo. Therefore, we concluded that WISP1 is increased in IBD and contributes to the proinflammatory cascades in the gut.

## 1. Introduction

Inflammatory bowel disease (IBD), which consists of Crohn's disease (CD) and ulcerative colitis (UC), is a chronic intestinal disorder with alternating relapse [1–3]. Although the pathological mechanisms of IBD remain unclear, it has been suggested that genetic changes and environmental factors contribute to the alterations of normal intestinal commensal flora, thus triggering excessive intestinal immune responses [4–6].

Various immune cell types infiltrate the inflamed mucosa of patients with IBD, and increasing evidence indicates that T cells, especially CD4^+^ T cells, play an important role in the tissue damage by secreting excessive proinflammatory cytokines [7, 8]. Previously, CD has been considered to be a Th1 cell-mediated disease with excessive production of tumor necrosis factor-*α* (TNF-*α*) and interferon-*γ* (IFN-*γ*) in the gut, whereas the Th2-like inflammatory cascades seem to be associated with the pathogenesis of UC [9]. However, recent studies focused on a new subtype of CD4^+^ T cells that produce IL-17, namely, Th17 cells, which is also an essential player in the pathogenesis of IBD [10]. It is also evident that IBD-related intestinal inflammation is maintained and enhanced by the deficiency of relative regulatory mechanisms, through decreased number of Foxp3^+^CD25^+^CD4^+^ T cells and IL-10 production [11]. Therefore, it is vital to restore the balance between proinflammatory and anti-inflammatory cascades as a potential valid therapeutic strategy to control excessive immune responses in IBD.

WNT1 inducible signaling pathway protein 1 (WISP1) is a secreted matricellular protein, which belongs to the CCN family. The CCN family of matricellular proteins is a complex family containing CCN1 to CCN6, namely, CCN1 (cysteine-rich protein 61, Cyr61), CCN2 (connective tissue growth factor, CTGF), CCN3 (nephroblastoma overexpressed gene, NOV), CCN4 (WNT1 inducible signaling pathway protein-1, WISP1), CCN5 (WISP2), and CCN6 (WISP3). It has been reported that WISP1 is associated with cellular responses, such as cell growth, survival, and differentiation [12]. In humans, WISP1 is expressed in various organs, including the lung, heart, pancreas, kidney, placenta, small intestine, and spleen, while low expression has been reported in the brain, skeletal muscle, liver, prostate, testis, and thymus [13]. WISP1 has been associated with cell proliferation, survival, and differentiation [14]. WISP1 expression has been detected in several cancers, such as hepatocellular carcinoma [15], colon adenocarcinomas [13], lung carcinoma [16], and breast cancer [17]. WISP1 has recently been reported to participate in UC and experimental colitis [18]. Recent studies have demonstrated that nitric oxide (NO), which was derived from the inducible NO synthase (iNOS), could induce WISP1 mRNA and protein expression through a *β*-catenin and CREB-dependent, but not Wnt-1-independent, pathway, thus aggravating intestinal inflammation [18]. However, the underlying mechanisms through which WISP1 regulates intestinal inflammatory cascades have not been fully elucidated yet.

In the current study, we investigated WISP1 mRNA and protein expression in inflamed mucosa and lamina propria mononuclear cells (LPMC) derived from patients with IBD and its potential role in regulating intestinal inflammation. We found that WISP1 expression was markedly elevated in inflamed mucosa and LPMC of patients with IBD. Tumor necrosis factor- (TNF-) *α* stimulation significantly upregulated WISP1 expression in IBD LPMC. Treatment with recombinant WISP1 (rWISP1) significantly enhanced secretion of the proinflammatory cytokines interferon- (IFN-) *γ*, IL-17, and IL-23 in IBD LPMCs. WISP1 expression was also found to be increased in 2,4,6-trinitrobenzenesulfonic acid- (TNBS-) induced experimental mouse colitis, a model similar to human Crohn's disease [19–21], and treatment with rWISP1 significantly exacerbated TNBS-induced colitis. Collectively, these data indicate that WISP1 could be a vital pathological molecule in intestinal inflammation.

## 2. Methods

### 2.1. Patients

Mucosal biopsies were obtained from inflamed and normal colonic mucosa of 44 patients with CD (21 males and 23 females, age 23–64 years), including 24 patients with active CD and 20 patients in remission; 39 patients with UC (22 males and 17 females, age 20–58 years), including 21 patients with active UC and 18 in remission; and 21 healthy controls (9 males and 12 females, age 25–52 years). Clinical characteristics of patients with IBD and healthy controls were displayed in Table 1. In another experiment, mucosal biopsies were taken from 13 patients with CD, 12 patients with UC, and 10 healthy controls. All patients were from Department of Gastroenterology, The Affiliated Hospital of Qingdao University (Qingdao, China). CD and UC were diagnosed according to clinical, radiological, and endoscopic examination as well as histological validation. All patients had not received immunosuppressive medications, hormone, biological agents, and blood transfusion therapy in recent one month and did not suffer from any other autoimmune diseases (such as ankylosing spondylitis, rheumatoid arthritis, and autoimmune hepatitis) and malignant diseases. There were no differences based on sex and age between groups. The severity of diseases was assessed according to Crohn's disease activity index (CDAI) for CD patients and Mayo scores for UC patients, respectively. Written informed consent was also obtained from all the patients before the study protocol, and the study protocol was approved by the local ethics committees.

### 2.2. Mice

Male BALB/c mice (8–10 weeks old) were kept in specific pathogen-free condition. All mice were raised under pathogen-free conditions in microisolator cages with filtered air and were fed standard pellet and autoclaved water. Male mice of 8–10 weeks (weight 19.8 ± 1.4 g) of age were used for experiments. All animal experiments were approved by the Animal Care and Use Committee at Qingdao University.

### 2.3. Peripheral Blood Mononuclear Cells (PBMCs) Isolation

Human PBMCs were isolated from EDTA-stabilized blood samples of healthy controls and IBD patients by Ficoll gradients (Life Technologies) as previously described with minor modifications [22]. In short, fresh peripheral blood was obtained from healthy controls and IBD patients and was mixed 1 : 1 with phosphate buffered saline (PBS, w/o Ca^2+^, Mg^2+^). The PBS-blood mixture was layered carefully onto Ficoll gradient and subsequently centrifuged at 800 ×g for 30 min, at room temperature (slow acceleration, no brake). The interphase layer was extracted and was diluted up to 30 mL with PBS. The cell contents were then centrifuged at 300 ×g for 10 min, at room temperature. Total PBMCs were stained with Trypan blue for determination of cell viability.

### 2.4. Isolation and Stimulation of Lamina Propria Mononuclear Cells (LPMCs)

LPMCs were isolated from mucosal specimens as previously described [23]. Briefly, mice were sacrificed and the colon was dissected, cut into 0.5 cm pieces, and washed thoroughly with cold PBS to remove all the stools. The colonic samples from IBD patients were obtained as indicated. Colonic tissues were incubated with 2 mM dithiothreitol (DTT) and 1 mM EDTA in PBS at 37°C for 20 min twice under gentle shaking to remove intestinal epithelial cells (IECs). Then, the tissues were digested in 10 mL 5% fetal bovine serum- (FBS-) RPMI-collagenase A (1 mg/mL, Sigma-Aldrich) at 37°C for 30 min. Lamina propria cells were purified via density gradient centrifugation with 40% and 80% Percoll-RPMI solution. LPMCs were collected from the interphase. LPMCs (5 × 10^5^/mL) from healthy controls were stimulated with anti-CD3 (5 *μ*g/mL) and anti-CD28 (2 *μ*g/mL) in the presence of TNF-*α*, IFN-*γ*, IL-6, IL-23, and IL-17A (20 ng/mL) for 48 h. Cells were then used for further analysis.

### 2.5. The TNBS-Induced Colitis Model

TNBS colitis model was induced as previously described [19–21]; BALB/c mice (11 mice per group) were divided into two groups and were fasted for 48 h with free access to drinking water to remove stool from the colon. Mice were then anesthetized with pentobarbital sodium, and a polyethylene catheter was inserted rectally up to the splenic flexure (5–8 cm from the anus). 2.0–2.5 mg TNBS dissolved in 50% ethanol was administrated to mice through the catheter, and then they were held in a headfirst position for at least 10 min. Only 50% ethanol was administered to mice in the control group. PBS or recombinant WISP1 (rWISP1) intraperitoneally at a dose of 1 mg/kg was administered to TNBS-induced mice. PBS or rWISP1 was administered to non-TNBS-treated mice as described above. The severity of colitis was monitored daily by recording parameters such as diarrhea, stool consistency, and rectal bleeding. Colonic tissues were dissected from mice, fixed in 10% paraformaldehyde overnight, embedded in paraffin, sectioned, and stained with H&E. The histological grading of colonic inflammation was graded from 0 to 3 as described previously [24–26].

### 2.6. RNA Extraction, RT PCR, and Quantitative Real-Time-PCR

Total RNA was extracted from cells and colonic samples using the RNeasy Kit from Qiagen (Valencia, CA, USA) according to the manufacturer's instructions. The total concentration of RNA was measured at 260 nm with a spectrophotometer (Beckman Coulter, Brea, CA, USA). First-strand complementary DNA (cDNA) synthesis was performed with 0.5 *μ*g of total RNA in a 10 *μ*L final volume containing 2 *μ*L PrimerScript RT Master Mix (Takara Bio Inc., Shiga, Japan) and 8 *μ*L of RNase-Free dH_2_O and total RNA. The reverse transcription procedure was carried out according to the manufacturer's instructions. Real-time PCR for glyceraldehyde-3-phosphate dehydrogenase (GAPDH), WISP1, IFN-*γ*, IL-17A, and IL-23 was performed using SYBR premix Ex Taq*™* (Takara) with a Step One Plus real-time PCR system (Applied Biosystems, USA), according to the manufacturer's instructions. GAPDH was used to normalize the gene expression of other mRNAs. The relative amount of each transcript was calculated according to the 2^−ΔΔCt^ method. All of the primers were synthesized by Invitrogen (Thermo Fisher Scientific). Primers sequence was as follows: hWISP1 forward 5′-AGGAACTGCATAGCCTACACA, reverse 5′-TGGTACACAGCCAGACACTTC, mWISP1 forward 5′-CGCCCGAGGTACGCAATAG, reverse 5′-GCAGTTGGGTTGGAAGGACT, hIL-17A forward 5′-AGATTACTACAACCGATCCACCT, reverse 5′-GGGGACAGAGTTCATGTGGTA, hIFN-*γ* forward 5′-TCGGTAACTGACTTGAATGTCCA, reverse 5′-TCGCTTCCCTGTTTTAGCTGC, and hIL-23 forward 5′-CTCAGGGACAACAGTCAGTTC, reverse 5′-ACAGGGCTATCAGGGAGCA.

### 2.7. Enzyme-Linked Immunosorbent Assay (ELISA)

Cell supernatants were collected and analyzed for hIL-17A content using a sensitive commercial ELISA kit (R&D Systems) according to the manufacturer's instructions.

### 2.8. Western Blot Analysis

Tissues and cells were harvested using iced-cold lysis buffer (Beyotime, catalog number P0013G). Protein concentration was determined using a bicinchoninic acid protein assay kit (Beyotime, catalog number P0011). Immunolabeling was detected using the Pierce ECL western blotting substrate (Thermo Fisher Scientific, catalog number 32109). The following antibodies and dilutions were used: human and mouse WISP1 antibody (1 : 1000) (R&D systems) and *β*-actin (1 : 10000) (Proteintech).

### 2.9. Statistical Analysis

All data were expressed as mean ± standard error of the mean (s.e.m.). Statistical analysis was performed using SPSS statistics version 14.0 (SPSS, Chicago, IL, USA). Differences between groups were compared using the Student *t*-test or one-way analysis of variance (ANOVA). A value of *p* < 0.05 was considered statistically significant.

## 3. Results

### 3.1. WISP1 Is Highly Increased in Inflamed Mucosa and LPMCs from Patients with IBD

**Figure 1:**
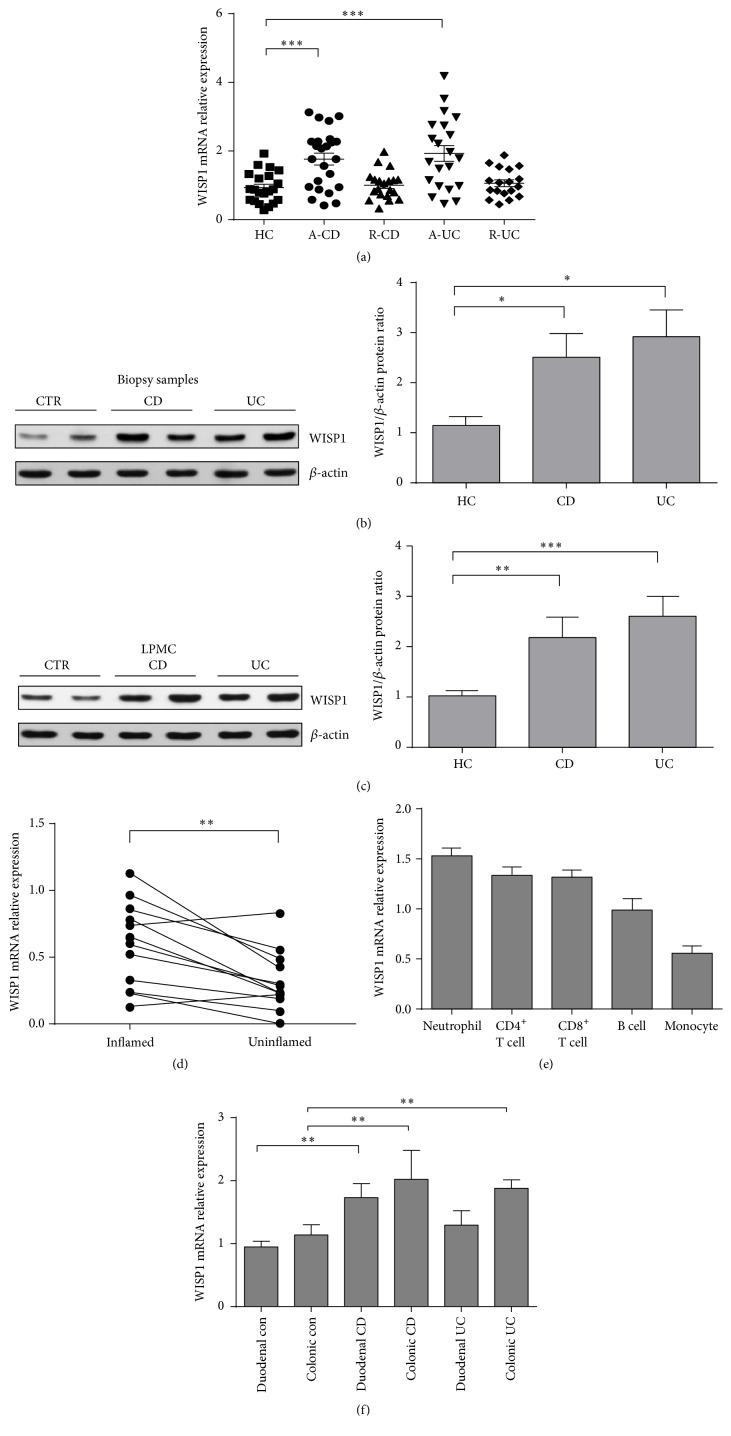
WISP1 is highly increased in inflamed mucosa and LPMCs of IBD patients. (a) qRT-PCR analysis of WISP1 in colonic samples from healthy controls (HC, *n* = 21), inflamed mucosa of active Crohn's disease (CD) patients (A-CD, *n* = 24), active UC patients (A-UC, *n* = 21), CD patients in remission (R-CD, *n* = 20), and UC patients in remission (R-UC, *n* = 18). (b) and (c) Representative western blots of WISP1 and *β*-actin in total proteins from colonic samples (b) and LPMCs (c) from HC (*n* = 13), CD patients (*n* = 16), and UC patients (*n* = 12). Right panel in (b) and (c) shows the quantitative analysis of WISP1/*β*-actin ratio. (d) Relative expression of WISP1 in intestinal mucosa from inflamed and uninflamed intestinal mucosa from the same patients with IBD (*n* = 13). (e) WISP1 expression in different subsets of immune cells. Peripheral blood B cells, CD4^+^ T cells, CD8^+^ T cells, monocytes, and neutrophils (1 × 10^6^ for each subset) were extracted from 10 healthy donors, and WISP1 expression was determined by qRT-PCR. (f) Mucosal biopsies were taken from 13 patients with CD, 12 patients with UC, and 10 healthy controls. The levels of mRNA for WISP1 were detected by qRT-PCR. Gene expression was normalized to GAPDH mRNA levels in each sample. (d) Paired Student's *t*-test and (a, b, c, and f) one-way ANOVA. ^**∗**^ *p* < 0.05, ^**∗∗**^ *p* < 0.01, and ^**∗∗∗**^ *p* < 0.001; data are expressed as mean ± s.e.m. for all samples.

Previous studies have demonstrated that nitric oxide (NO) induces WISP1 mRNA and protein expression in UC [18]; we then sought to examine whether WISP1 expression is also upregulated in the inflamed mucosa of patients with CD and whether WISP1 expression was altered in IECs or LPMCs. WISP1 expression was found to be markedly increased in the inflamed mucosa from both CD and UC patients compared with that from healthy controls (Figure 1(a)). Furthermore, we examined WISP1 protein expression in whole biopsy specimens of IBD and controls by western blotting. Western blot analysis showed that WISP1 was expressed in healthy controls and IBD samples (Figure 1(b)). However, its expression was significantly increased in both UC and CD samples compared with healthy controls (Figure 1(b)). Further analysis of WISP1 in total proteins extracted from LPMCs samples confirmed the increased expression of WISP1 in IBD compared with healthy controls (Figure 1(c)). We next compared WISP1 expression in the inflamed and unaffected mucosa from the same patients with IBD and found that higher levels of WISP1 were present in the inflamed mucosa compared with the unaffected intestinal mucosa from the same patients (Figure 1(d)). To determine the expression of WISP1 in subsets of immune cells, peripheral blood CD4^+^, CD8^+^ T cells, B cells, monocytes, and neutrophils were isolated from 15 healthy donors, and WISP1 was found to be expressed at relatively high levels in neutrophils, CD4^+^ T cells, and CD8^+^ T cells (Figure 1(e)). To further investigate WISP1 relative expression in different location of colons from IBD patients and healthy controls, we used RNA samples from duodenal healthy controls, CD and UC patients, and colonic healthy controls to verify mRNA relative expression of WISP1. The results showed that WISP1 was markedly increased in duodenal and colonic CD patients compared with healthy controls. WISP1 was also significantly upregulated in colonic UC patients compared with healthy controls. However, there was no significance between the transcripts of duodenal UC and healthy controls.

### 3.2. TNF-*α* Upregulates WISP1 Expression in IBD LPMC

**Figure 2:**
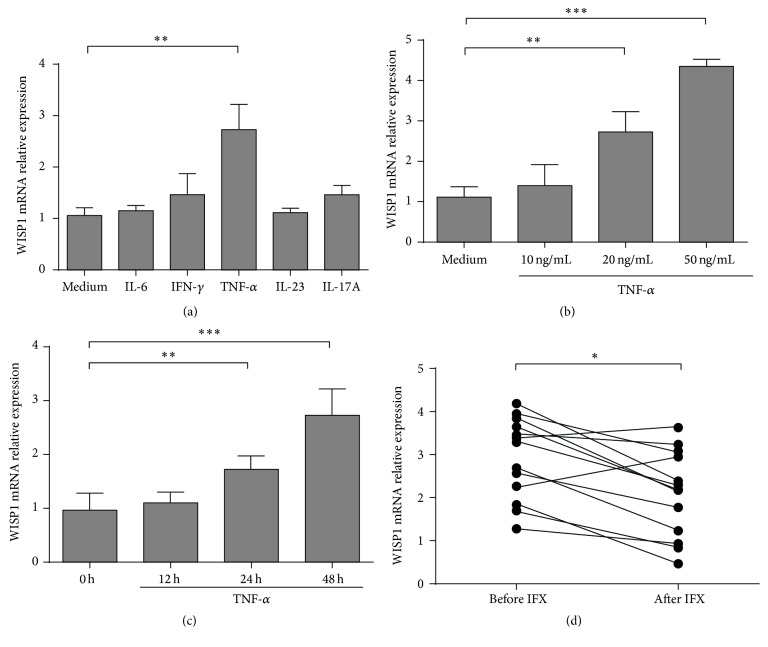
Tumor necrosis factor-*α* (TNF-*α*) upregulates WISP1 expression. (a) LPMCs (5 × 10^5^/mL) from healthy controls were stimulated with anti-CD3 (5 *μ*g/mL) and anti-CD28 (2 *μ*g/mL) in the presence of TNF-*α*, IFN-*γ*, IL-6, IL-23, and IL-17A (20 ng/mL) for 48 h, and WISP1 relative expression was determined by qRT-PCR. (b) LPMCs were stimulated with anti-CD3 (5 *μ*g/mL) and anti-CD28 (2 *μ*g/mL) in the presence of different doses of TNF-*α* (10 ng/mL, 20 ng/mL, and 50 ng/mL) for 48 h. WISP1 relative expression was determined by qRT-PCR. (c) LPMCs were stimulated with anti-CD3 (5 *μ*g/mL) and anti-CD28 (2 *μ*g/mL) in the presence of TNF-*α* (20 ng/mL) and cultured for 12 h, 24 h, and 48 h. WISP1 relative expression was determined by qRT-PCR. (d) Colonic biopsies were obtained from CD patients prior to and after IFX treatment. WISP1 relative expression was determined by qRT-PCR. Representative results from three independent experiments are shown. (a, b, and c) One-way ANOVA and (d) Student's *t*-test. ^*∗*^ *p* < 0.05, ^*∗∗*^ *p* < 0.01, and ^*∗∗∗*^ *p* < 0.001; data are expressed as mean ± s.e.m. for all samples.

The confirmation that WISP1 expression is upregulated in IBD prompted us to explore whether it is positively regulated by some proinflammatory cytokines [8]. As accumulating evidence has shown that increased proinflammatory cytokines such as TNF-*α*, IL-23, and IL-6 are detected in inflamed mucosa of IBD patients [27], we explored whether these cytokines could regulate WISP1 expression in IBD LPMCs. LPMCs (5 × 10^5^/mL) from healthy controls were stimulated with anti-CD3 (5 *μ*g/mL) and anti-CD28 (2 *μ*g/mL) in the presence of TNF-*α*, IFN-*γ*, IL-6, IL-23, and IL-17A (20 ng/mL) for 48 h. Cells were then used for qRT-PCR analysis. Interestingly, WISP1 transcripts were upregulated in LPMCs from healthy controls stimulated by TNF-*α*, but not by IFN-*γ*, IL-6, IL-23, and IL-17A (Figure 2(a)). Furthermore, we found that TNF-*α* upregulated WISP1 expression in a dose- (Figure 2(b)) and time-dependent (Figure 2(c)) manner. To further examine whether the increased TNF-*α* contributes to the upregulation of WISP1 in IBD patients, we detected WISP1 relative expression in intestinal mucosa from CD patients prior to and after treatment with anti-TNF-*α* mAb (IFX). Eleven CD patients were recruited and were treated with IFX at weeks 0, 2, and 6 as previously described [28]. Interestingly, we found that WISP1 expression was significantly decreased in the inflamed mucosa of CD patients in the remission group after IFX induction compared with that before IFX treatment (Figure 2(d)). Collectively, these data suggested that TNF-*α* was able to upregulate WISP1 expression in IBD patients and treatment with IFX could negatively regulate WISP1 expression in the intestine.

### 3.3. WISP1 Facilitates the Expression of Proinflammatory Cytokines in IBD LPMCs

**Figure 3:**
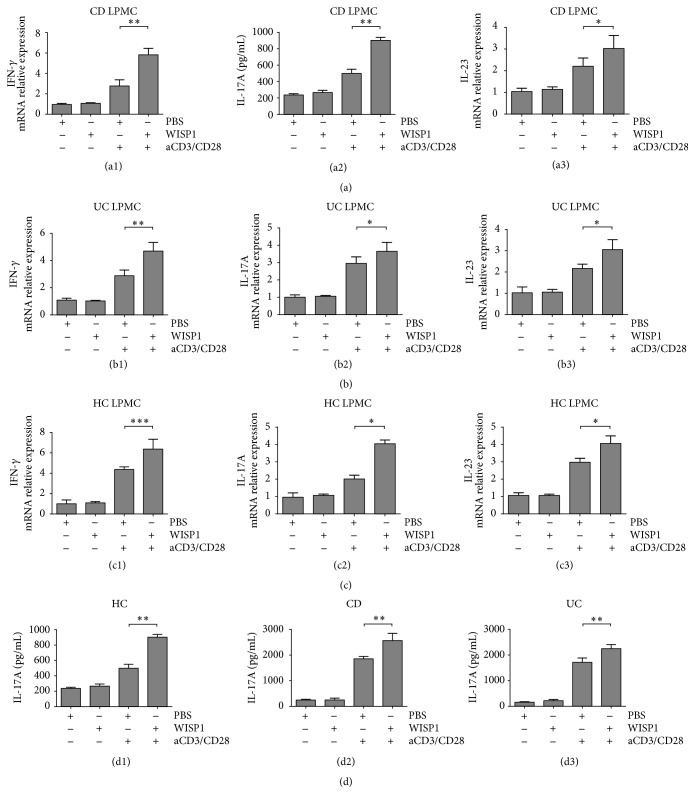
WISP1 facilitates the expression of proinflammatory cytokines in IBD LPMCs. LPMCs were isolated from CD patients (a), UC patients (b), and healthy controls (c). Cells were either pretreated with medium or activating anti-CD3/CD28 antibody-coated beads for 6 h. Cells were then incubated with rWISP1 (20 *μ*g/mL) or PBS (vehicle) for another 6 h before RNA extraction and further qRT-PCR analysis. qRT-PCR analysis of IFN-*γ*, IL-17A, and IL-23 was performed in LPMCs from healthy controls (a1, a2, and a3), UC patients (b1, b2, and b3), and CD patients (c1, c2, and c3). Supernatant of the above samples was collected and ELISA of IL-17A was performed ((d1) for healthy controls, (d2) for CD patients, and (d3) for UC patients). One-way ANOVA was performed. ^*∗*^ *p* < 0.05, ^*∗∗*^ *p* < 0.01, and ^*∗∗∗*^ *p* < 0.001; data are expressed as mean ± s.e.m. for all samples.

Next, we investigated whether increased expression of WISP1 could affect the expression of proinflammatory cytokines in IBD LPMCs. Our studies focused on the IFN-*γ*, IL-17A, and IL-23, which belong to the Th1/Th17 cytokines in IBD [29–31]. Real-time PCR showed that rWISP1 enhanced IFN-*γ*, IL-17A, and IL-23 transcripts in LPMCs from CD patients induced by anti-CD3/CD28 compared with those treated with control medium (Figure 3(a)). No significance was detected between groups without anti-CD3/CD28 stimulation. In LPMCs from UC patients (Figure 3(b)) and healthy controls (Figure 3(c)), rWISP1 also facilitated IFN-*γ*, IL-17A, and IL-23 relative expression. ELISA showed that rWISP1 enhanced the production of IL-17A in LPMCs from healthy controls, CD patients, and UC patients (Figure 3(d)). Together, these data suggested that WISP1 enhances expression of proinflammatory cytokines, thus contributing to the aggravation of colitis.

### 3.4. WISP1 Expression Is Upregulated in TNBS-Induced Colitis

**Figure 4:**
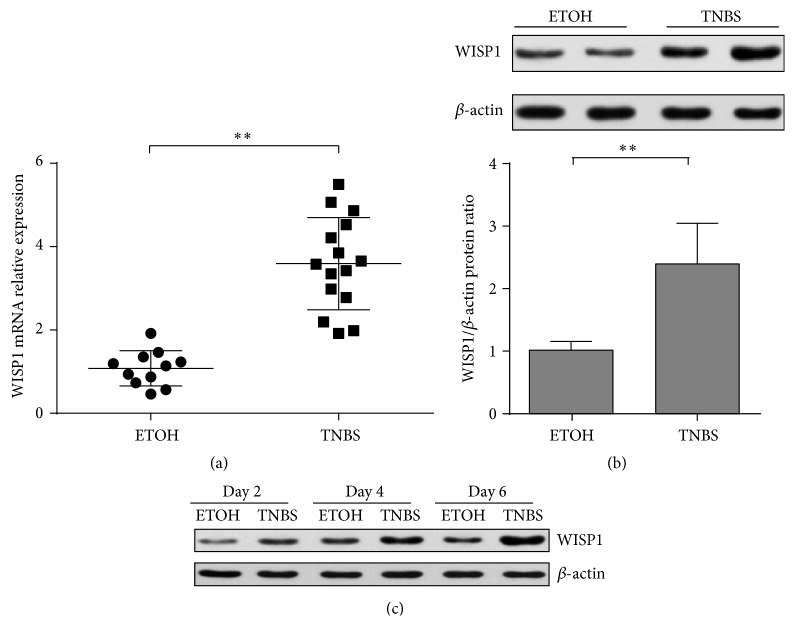
WISP1 was upregulated in TNBS-induced colitis. (a) WISP1 mRNA relative expression was determined in colonic samples from ethanol-treated mice (*n* = 11) and TNBS-treated mice (*n* = 15) by (qRT-)PCR. (b) Western blot analysis of WISP1 expression in total proteins from colonic samples from ethanol-treated mice (*n* = 8) and TNBS-treated mice (*n* = 6). Lower panel in (b) shows the quantitative analysis of WISP1/*β*-actin ratio. (c) Representative western blot analysis of WISP1 expression in total proteins from colonic samples from ethanol-treated mice and TNBS-treated mice at days 2, 4, and 6 after colitis induction, respectively. Representative results from three independent experiments are shown. Student's *t*-test was performed. ^*∗∗*^ *p* < 0.01 data are expressed as mean ± s.e.m. for all samples.

To further determine whether WISP1 was also upregulated in experimental colitis, 2,4,6-trinitrobenzenesulfonic acid- (TNBS-) induced colitis was applied to verify the mRNA and protein expression of WISP1 in colitis. WISP1 mRNA relative expression was significantly increased in the colons of TNBS-treated mice compared with ethanol-treated controls (Figure 4(a)). Western blot analysis of WISP1 indicated that WISP1 expression was detected in control and TNBS colitis mice (Figure 4(b)). Next, we examined WISP1 expression at different time points of TNBS colitis model and found that upregulation of WISP1 was observed at early time points and persisted during the whole model (Figure 4(c)).

### 3.5. Treatment of rWISP1 Aggravates TNBS-Induced Colitis

**Figure 5:**
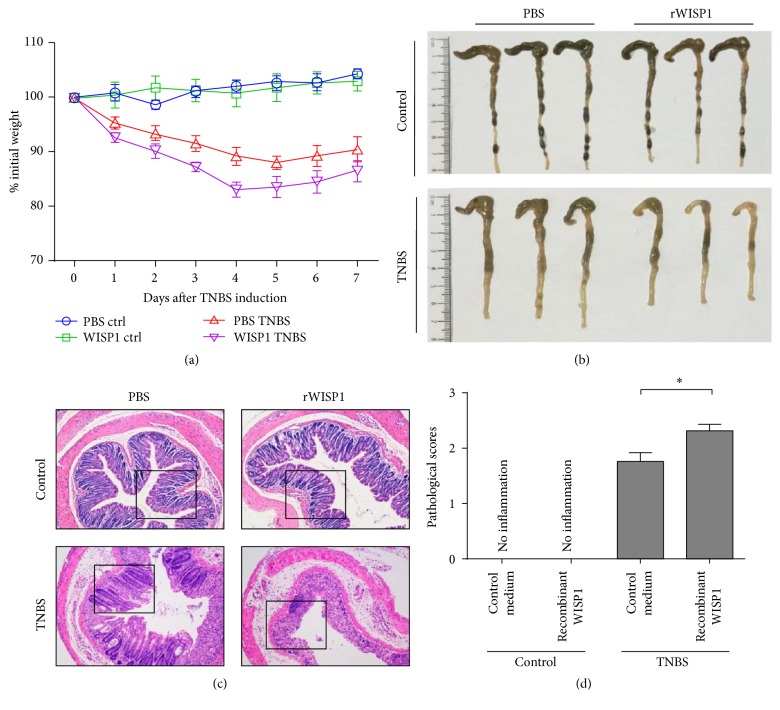
Treatment of rWISP1 aggravates TNBS-induced colitis in vivo. (a) BALB/c mice were TNBS colitis induced as indicated. rWISP1 or control medium intraperitoneally daily starting at the beginning of the TNBS induction till the end of the model was administered to two groups of TNBS-induced mice. rWISP1 or control medium as indicated above was administered to two groups of non-TNBS-treated mice. The changes in body weight during the whole colitis model are expressed as a percentage of the original weight at the start of the experiments. (b) Representative morphology of the intestine on day 7 after TNBS treatment. (c) Representative H&E staining of the colons (original magnification ×200). (d) Pathological scores were evaluated during the whole colitis model. Student's *t*-test was performed. ^*∗*^ *p* < 0.05; data are expressed as mean ± s.e.m. for all samples.

To further investigate WISP1 in the pathogenesis of experimental colitis, we sought to determine whether administration of rWISP1 could aggravate intestinal inflammation in vivo. TNBS-induced colitis was induced in BALB/c mice by intracolonic administration with TNBS, and rWISP1 was administered intraperitoneally daily as indicated. Control medium (PBS) was administered to control (non-TNBS-administered) mice and rWISP1-administered mice served as negative controls. Clinical characteristics, such as body weight, stool consistency, and rectal bleeding, were recorded daily. On day 7 of TNBS-induced colitis, mice were sacrificed and colonic samples were collected for further studies. Treatment with rWISP1 significantly aggravated murine TNBS-induced colitis, as shown by markedly increased weight loss (Figure 5(a)), shorter colon length (Figure 5(b)), higher disease activity index, and higher levels of pathological indices (Figures 5(c) and 5(d)) compared with those treated with control medium. However, no significant changes were observed in non-TNBS-induced mice treated with either control medium or rWISP1 (Figures 5(a)–5(d)). Collectively, these data suggested that rWISP1 administration aggravates TNBS-induced colitis in vivo, further supporting a role for WISP1 in intestinal inflammation pathology.

## 4. Discussion

Abundant advances have been achieved in exploring the pathogenesis of IBD. It is widely accepted that the pathogenesis of IBD includes intestinal microbiota, environmental factors, disruption of intestinal barrier function, and dysregulation of immune responses in the gut [5, 8, 30, 32]. Recent studies focused on the dysregulation of intestinal immune responses. An efficient host immune system is required for maintaining the delicate balance in this host-bacteria interaction and for inhibiting access of bacteria to the lamina propria (LP) of the gut. Various factors participate in the disruption of intestinal barrier function through downregulation of tight junctions (TJ) on intestinal epithelial cells, thus contributing to the access of bacterial antigens to LP [33]. Upon recognition and presentation of antigens through a vast diversity of receptors, capable of recognizing a huge variety of antigens and eliciting specific and memory responses, such as pattern recognition receptors (PRR) in innate immune cells, adaptive immune systems are activated, thus contributing to various kinds of immune cascades, including neutrophil recruitment, macrophage, and CD4^+^ T cell activation [34], with multiple kinds of proinflammatory cytokine production such as TNF-*α*, IFN-*γ*, IL-17A, and IL-23 [32].

The WNT1 inducible signaling pathway protein has 3 isoforms, namely, WISP1–WISP3, identified by Pennica et al. [13]. They were reclassified to the CCN protein family and renamed CCN4/WISP1, CCN5/WISP2, and CCN6/WISP3 [35]. WISP1 has been demonstrated to participate in cancer, such as hepatocellular carcinoma, colon adenocarcinomas, lung carcinoma, and breast cancer [15, 16], and other diseases such as osteoarthritis and lung fibrosis [36, 37]. The biological function of CCN family has been linked to wound healing [38] and organ fibrosis [12]. However WISP1 expression was associated with multiple biological processes such as cell proliferation, survival, and cell differentiation. Recent studies have demonstrated that WISP1 treatment enhanced proliferation of hBMSC in vitro and TGF-*β* or BMP2 was necessary to induce differentiation of hBMSC into osteoblasts [39]. Another study demonstrated that WISP1 increased cell proliferation but not differentiation into chondrocytes in prechondrocytes [40]. Previous studies have demonstrated that NO can induce WISP1 expression in UC [18]. However, the mechanisms underlying how WISP1 mediated intestinal inflammation in IBD patients and experimental colitis remain unclear.

In our study, we investigated the expression and pathological roles of WISP1 in the intestine of IBD patients and experimental colitis. Here we observed a significant increase in WISP1 mRNA expression in colonic biopsies and LPMCs from both CD and UC patients compared with those from healthy controls. Western blot analysis further confirmed the upregulation of WISP1 protein in IBD, especially in neutrophils, CD4^+^ T cells, and CD8^+^ T cells. In human colonic biopsies from IBD patients, WISP1 relative expression was found to be markedly increased in inflamed colons compared with uninflamed tissues. These results indicate that WISP1 expression is upregulated in IBD and might contribute to the pathogenesis of IBD. However, the mechanisms why WISP1 was upregulated in colonic biopsies and LPMCs were still not fully elucidated. WISP1 could be regulated at transcriptional, posttranscriptional, and posttranslational levels by various factors. Our study also focused on the regulatory factors that were involved in WISP1 regulation in the human intestine. We sought to investigate whether some proinflammatory cytokines that contributed to the pathogenesis of IBD could upregulate WISP1 expression. Indeed, our data indicated that TNF-*α* was able to induce WISP1 expression in a time- and dose-dependent manner. Furthermore, WISP1 expression was decreased in IBD patients after treatment of IFX compared with that before IFX administration. Next, we would like to investigate how WISP1 contributes to inflammatory cascades in IBD. LPMCs from IBD patients and healthy controls were isolated and treated with rWISP1. Interestingly, WISP1 significantly enhanced production of proinflammatory cytokine such as IL-17A, IFN-*γ*, and IL-23. Collectively, these data indicated that TNF-*α* enhanced WISP1 expression, thus contributing to IL-17A, IFN-*γ*, and IL-23 expression in IBD LPMCs.

Consistent with the data in human studies, we also observed that WISP1 mRNA and protein expression was upregulated in TNBS-induced experimental colitis and WISP1 expression was increased at early time points of TNBS colitis model and persisted till the end. To further confirm the proinflammatory role of WISP1 in the pathogenesis of IBD and whether administration of rWISP1 could aggravate TNBS-induced colitis, we administered rWISP1 into BALB/c mice during TNBS-induced colitis and found that rWISP1 exacerbated intestinal inflammation, characterized by more weight loss, shorter colon length, and severe clinical features as well as higher pathological scores. Collectively, these data further indicated the proinflammatory role of WISP1 in colitis in vivo.

## 5. Conclusion

In conclusion, our study demonstrated that WISP1 expression is upregulated in the gut of IBD patients and TNBS-induced experimental colitis. Administration of rWISP1 aggravates proinflammatory cytokine production in LPMCs from IBD patients. Furthermore, administration of rWISP1 exacerbates TNBS-induced colitis in vivo. Our study shed a new light on the role of WISP1 in the pathogenesis of IBD and indicates that targeting against WISP1 might be a novel approach for treatment of IBD.

## Figures and Tables

**Table 1:** Clinical characteristics of patients with IBD and controls.

	Biopsy samples		
	Con	CD	UC
*Numbers of patients*	21	44	39
*Age (y)*	35.7 ± 8.9	44.2 ± 16.8	39.6 ± 12.3
*Gender*			
Male	9	21	22
Female	12	23	17
*Disease duration (month)*		41.2 ± 16.7	44.2 ± 23.4
*Current therapy*			
5-Aminosalicylates		34	33
Immunosuppressants		0	0
Biologics		0	0
Nutritional therapy		4	0
*Disease extent (UC)* ^*∗*^			
E1			13
E2			16
E3			10
*Disease location (CD)* ^*∗*^			
L1		8	
L2		16	
L3		20	
L4		0	

^*∗*^According to the Montreal classification system.
